# Environmental DNA reveals the Salamander Mussel *Simpsonaias ambigua* alive in Illinois, USA, after a century in obscurity

**DOI:** 10.1002/ecy.70145

**Published:** 2025-07-02

**Authors:** Sarah A. Douglass, Savanna Palmer, Ashleigh R. McCallum, Olivia P. Reves, Hayley A. Robinson, Allison J. Rutledge, Jordan H. Hartman, Eric R. Larson, Mark A. Davis

**Affiliations:** ^1^ Illinois Natural History Survey, Prairie Research Institute University of Illinois Urbana‐Champaign Champaign Illinois USA; ^2^ Department of Natural Resources and Environmental Sciences University of Illinois Urbana‐Champaign Urbana Illinois USA; ^3^ School of Integrative Biology University of Illinois Urbana‐Champaign Urbana Illinois USA

**Keywords:** artificial structures, conventional surveys, endangered species, environmental DNA, freshwater mussel, habitat use, salamander

Anthropogenic stressors are driving global biodiversity losses (Tulloch et al., 2020), and have fallen particularly hard on freshwater mussels, rendering them among the most imperiled faunal groups (Aldridge et al., 2023; Brian & Aldridge, 2023; Gallardo et al., 2018). Freshwater mussel loss has been caused by numerous factors including increased nutrient loading, pollution, invasive species introductions, habitat loss (both instream and adjacent riparian zones), and degradation, all often acting synergistically (Aldridge et al., 2023; Ferreira‐Rodriguez et al., 2019; Nakamura et al., 2023). Consequently, imperiled freshwater mussels are increasingly granted formal protection (Haag & Williams, 2014). For example, of the approximately 300 native freshwater mussel species of North America, over 70% are considered endangered, threatened, or of special concern in many states (Williams et al., 1992). There are 96 mussel species (excluding experimental populations) formally protected under the United States Endangered Species Act (50 CFR 17.11) as listed by the environmental conservation online system (ECOS) website (https://ecos.fws.gov/), and 18 species protected under Canada Species at Risk Act (S.C. 2002, c. 29) as listed in the species at risk public registry (https://www.canada.ca/en/environment-climate-change/services/species-risk-public-registry.html).

The Salamander Mussel *Simpsonaias ambigua* (Say 1825) is one such freshwater mussel. A diminutive, nondescript bivalve in the family Unionidae, the Salamander Mussel is unusual, given that the only known host for its larvae is the Mudpuppy *Necturus maculosus* (Rafinesque 1818), a fully aquatic salamander species. Salamander Mussels are found in lentic and lotic habitats, with their microhabitat typically consisting of large, flat stones where the mussel may be more likely to co‐occur with its salamander host (Parmalee & Bogan, 1998). The historical range of the Salamander Mussel extended across the United States in Arkansas, Illinois, Indiana, Iowa, Kentucky, Michigan, Minnesota, Missouri, New York, Ohio, Pennsylvania, Tennessee, West Virginia, Wisconsin and in Canada in the province of Ontario (NatureServe, 2024). Considered Vulnerable on the IUCN Red List (Bogan et al., 2017) and Critically Imperiled (G1G2) by NatureServe (NatureServe, 2024), the Salamander Mussel has been granted state‐level protections in several states, endangered status under Canada's Species at Risk Act (Morris & Burridge, 2006), and is currently proposed for federal listing as endangered under the United States Endangered Species Act (U.S. Fish and Wildlife Service, 2023).

In Illinois, no live Salamander Mussels have been documented for well over a century, since Baker (1906) listed presumably live specimens from a handful of records, despite intense survey efforts (Cummings et al., 2002; Douglass & Stodola, 2014). Both the mussel and its salamander host remain difficult to detect via conventional survey methods due to their unique microhabitat preferences.

Emerging technologies and methodologies such as environmental DNA (eDNA) methods have shown particular promise as a survey tool to detect rare freshwater species (Takahashi et al., 2023). Detecting threatened or endangered freshwater mussels via eDNA has resulted in successful live captures of mussel species with atypical habitat requirements, such as sheltering under rocks (Lor et al., 2020; Porto‐Hannes et al., 2023) and helped assess the distributions of rare species (Johnson et al., 2025; Prié et al., 2023; Roderique, 2018). Environmental DNA methodologies can be used to identify eDNA decay rates, seasonal activity, and, potentially, abundances of a species to help inform conventional sampling strategies to guide conservation interventions (Sansom & Sassoubre, 2017; Schmidt et al., 2021; Wacker et al., 2019). Across various rare freshwater taxa, eDNA continues to be a powerful tool for identifying extant populations and informing conventional sampling efforts for species that are extirpated, possibly extinct, or have unknown status (Janosik et al., 2021; Oliveira Carvalho et al., 2024). Furthermore, in this study, positive eDNA results facilitated a targeted approach to conventional Salamander Mussel surveys.

On 24 June 2024, we conducted eDNA sampling at eight sites in the Sangamon River, Champaign County, Illinois, United States, targeting both Mudpuppy and the Salamander Mussel. Two historical shell records exist for Salamander Mussels in the Sangamon River in the Illinois Natural History Mollusk Collection (INHS 12115, INHS 25013), as well as several records for Mudpuppy (Illinois Natural History Survey – Amphibian and Reptile Collection, 2025). At each site, we collected three 1‐L water samples, taken facing upstream at right, center, and left channel (Figure 1a). All water samples were collected at the surface with 1‐L high‐density polyethylene (HDPE) bottles (Nalgene). A 1‐L negative control, first filled with distilled water (DI) at the laboratory, was also taken to each site to account for potential contamination. Specifically, the blank was removed from the cooler, the top removed and held open for 60 s, returned to the cooler, on ice, and stored with subsequent field samples. All samples were handled per Curtis et al. (2021). Nitrile gloves were changed, and waders were bleached between each site.

**FIGURE 1:**
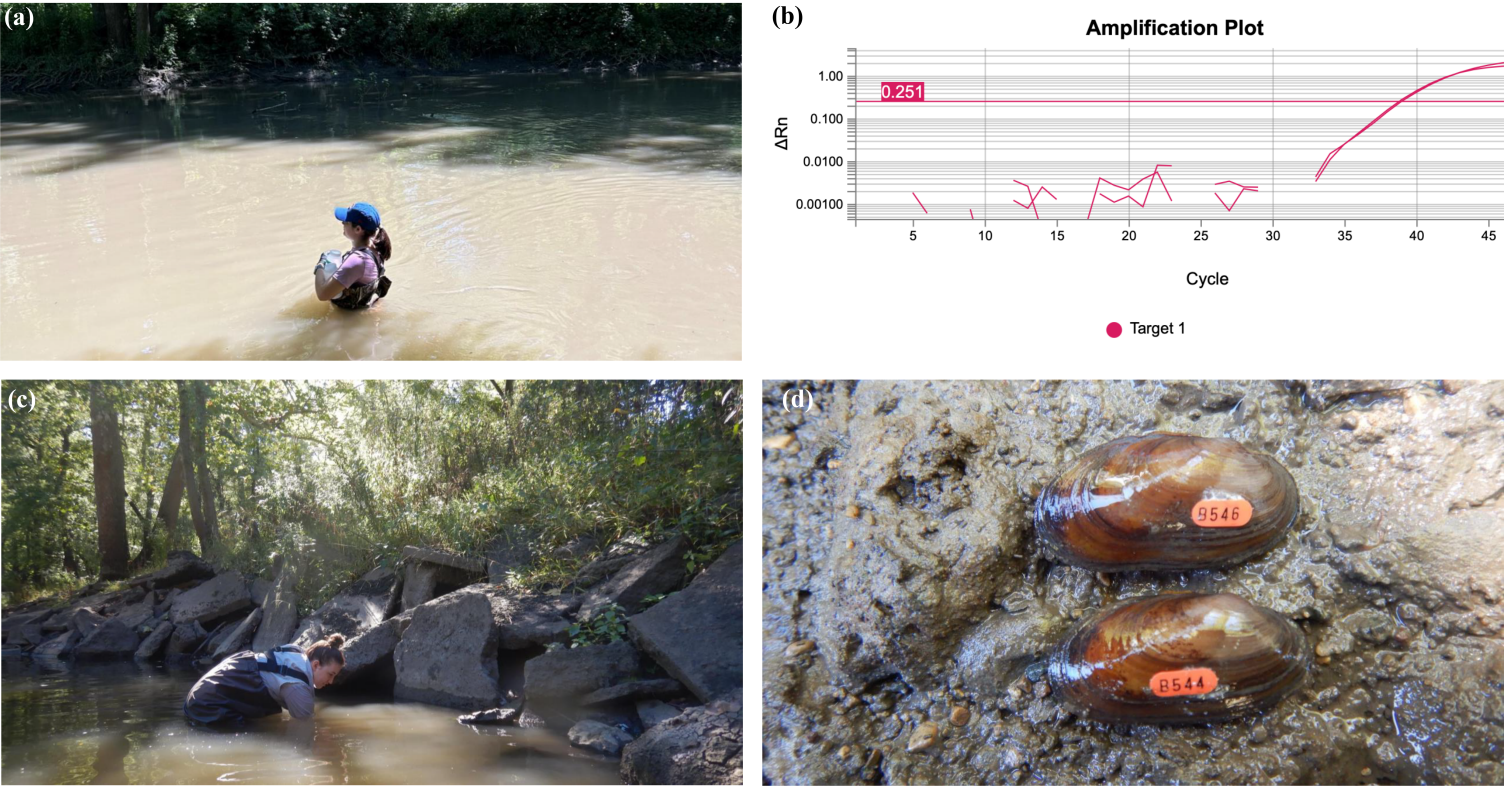
(a) Water collection for Salamander Mussel and Mudpuppy environmental DNA in the Sangamon River in central Illinois on 24 June 2024. Photograph: Savanna Palmer; (b) ΔRn is the magnitude of normalized fluorescence signal, relative to the baseline fluorescence. 0.251 represents the critical threshold (CT) and amplification above CT represents a positive amplification of a technical replicate for Salamander Mussel eDNA in qPCR analysis; (c) artificial habitat where live Salamander Mussels were encountered on 2 October 2024 in the Sangamon River. Photograph: Ashleigh McCallum; (d) two specimens of Salamander Mussel with unique ID shellfish tag on 2 October 2024. Photograph: Sarah Douglass.

After collection, samples were immediately stored in the dark and on ice in coolers, and within 6 h of collection were filtered through a 1.0 μm cellulose nitrate membrane (Whatman, General Electric Healthcare, Chicago, IL) filter. Filters were placed into Cetyltrimethylammonium bromide (CTAB) buffer solution and stored at room temperature for 2 weeks to isolate eDNA. We then used a phenol‐chloroform‐isoamyl alcohol extraction protocol that included an extraction negative control (Renshaw et al., 2015). Extracted eDNA was then subjected to quantitative polymerase chain reaction (qPCR) using previously designed, optimized, and validated assays for both Salamander Mussel (Porto‐Hannes et al., 2023) and Mudpuppy (Collins et al., 2019), per the conditions stipulated in those manuscripts, but using TaqMan Environmental Master Mix (Applied BioSystems). For each sample, three technical replicates were run per assay, and, in addition to field and extraction negative controls, three no template controls (NTC) were run on each plate. Finally, we ran an eight‐point dilution series consisting of synthetic Salamander Mussel and Mudpuppy DNA (i.e., gBlocks, Integrated DNA Technologies) as positive controls. We considered a sample to be positive if one technical replicate was positive, amplifying above a critical threshold value of 0.251 (Figure 1b.). Sample filtration, eDNA extraction, and qPCR preparations were all conducted in sterile hoods in a dedicated clean room, physically isolated from the main PCR lab. In between processes, hoods, benchtop surfaces, instruments, pipettes, and consumables were sterilized with 10% bleach for 10% contact time and subjected to 20 min of ultraviolet radiation to minimize contamination. In addition, nitrile gloves were changed frequently throughout these processes.

Our screening of eight sites in the Sangamon River yielded eDNA detections of exclusively Mudpuppy at three sites, while both Mudpuppy and Salamander Mussel were detected at one site. At that site, Mudpuppy was detected from both the left and center channel samples, while Salamander Mussel was detected from both the left and right channel samples. All field and extraction negative controls, as well as all NTC, failed to amplify, while all positive controls were successfully amplified.

On the morning of 2 October 2024, a seven‐person hour survey was conducted at the site that yielded the eDNA detections for both Mudpuppy and Salamander Mussel. The survey targeted the preferred habitat of the Salamander Mussel and focused on large, flat concrete material (hereafter, rocks) along the streambank of an outer bend in the river and adjacent bridge pool (Figure 1c.). We collected 12 Salamander Mussels, in groups of 1–4 individuals from under five rocks with diameters of <1 m × 1 m in length and width. Substrate from all areas with live individuals included sand and fine gravel mixed with a modest layer of fine silt. One additional rock with recently dead individuals had excessive silt with detritus material over fine gravel and likely became anoxic due to recent low water levels at these rocks closest to the bank. Individuals were kept within groups and subsequently measured, aged, checked for gravidity, swabbed for genetic analysis, and tagged with a unique shellfish ID tag (Figure 1d). Seven Salamander Mussels appeared gravid when examined internally with a small speculum. All groups were returned to their respective rocks (Porto‐Hannes et al., 2021). Dead shells and two live individuals were vouchered and accessioned into the Illinois Natural History Survey – Mollusk Collection (2025) as lot INHS 95333.

Our observations illustrate the immense value of eDNA to concentrate efforts and more precisely deploy taxonomic expertise for our rarest species. In this case, positive eDNA detections prompted a targeted conventional search that yielded live Salamander Mussel for the first time in over a century in Illinois. Illinois has among the most comprehensive biodiversity inventories in the world (Smith, 1969), but conventional sampling has failed to detect live Salamander Mussels. Furthermore, artificial habitat with slab‐type concrete materials originally installed for streambank stabilization on the outer bank of the riverbend created the right habitat for decades that ensured the persistence of the Salamander Mussel and Mudpuppy population in the Sangamon River (Figure 1c). While much of the riparian areas in the upper Sangamon River have remained forested, the watershed is dominated by agricultural land use. Land use in central Illinois has changed across time driven by anthropogenic factors, and, as a result, suspended sediments continue to increase (Yu & Rhoads, 2018). These factors may change habitats instream and possibly limit preferred structure options for the Salamander Mussel (Watson et al., 2001). The Sangamon River is typically turbid and dominated by sand, coarse gravel substrates in the thalweg, woody debris, and with silt and detritus material along the banks (S. Douglass, pers. obs). Mudpuppy are known to utilize a variety of structures and cover objects (e.g., woody debris, tree roots); can Salamander Mussels inhabit these shelter types as well? This facet of Salamander Mussel natural history requires further research. Mudpuppy conservation efforts in surrounding states have included adding artificial structures with successful habitation of Mudpuppy and an instance of recruitment of one live Salamander Mussel (U.S. Fish and Wildlife Service, 2023). This research provides useful demographic information from a wild population and highlights successful artificial habitat use to the collective information on the Salamander Mussel. Finding live Salamander Mussels in Illinois contributes to our knowledge of the distribution of a rare species whose fate is intimately tied to its host, Mudpuppy, which also has insufficient distributional data in Illinois. In this case, applying eDNA methodologies in tandem for both species will serve to update distributions and status assessments, helping to inform important conservation planning at state and federal levels (U.S. Fish and Wildlife Service, 2023).

## CONFLICT OF INTEREST STATEMENT

The authors declare no conflicts of interest.

## Data Availability

Data (Davis, 2025) are available in the Illinois Data Bank, University of Illinois Urbana‐Champaign at https://doi.org/10.13012/B2IDB-5667464_V1.
